# Laser Emission at 675 nm: Molecular Counteraction of the Aging Process

**DOI:** 10.3390/biomedicines12122713

**Published:** 2024-11-27

**Authors:** Lorenzo Notari, Laura Pieri, Francesca Cialdai, Irene Fusco, Chiara Risaliti, Francesca Madeddu, Stefano Bacci, Tiziano Zingoni, Monica Monici

**Affiliations:** 1 ASA Campus Joint Laboratory, ASA Research Division, Department of Experimental and Clinical Biomedical Sciences “Mario Serio”, University of Florence, 50139 Florence, Italy; lorenzo.notari@unifi.it (L.N.); francesca.cialdai@unifi.it (F.C.); chiara.risaliti@unifi.it (C.R.); monica.monici@unifi.it (M.M.); 2 El.En. Group, 50041 Calenzano, Italy; l.pieri@deka.it (L.P.); f.madeddu@elen.it (F.M.); t.zingoni@elen.it (T.Z.); 3 Research Unit of Histology and Embryology, Department of Biology, University of Florence, 50121 Florence, Italy; stefano.bacci@unifi.it

**Keywords:** 675 nm laser, extracellular matrix molecules, skin rejuvenation

## Abstract

Background/Objectives: Many lasers applied in skin rejuvenation protocols show emissions with wavelengths falling in the red or near-infrared (NIR) bands. To obtain further in vitro data on the potential therapeutic benefits regarding rejuvenation, we employed a 675 nm laser wavelength on cultured human dermal fibroblasts to understand the mechanisms involved in the skin rejuvenation process’s signaling pathways by analyzing cytoskeletal proteins, extracellular matrix (ECM) components, and membrane integrins. Methods: Normal human dermal fibroblasts (NHDFs) were irradiated with a 675 nm laser 24 h after seeding, and immunofluorescence microscopy and Western blotting were applied. Results: The results demonstrate that the laser treatment induces significant changes in human dermal fibroblasts, affecting cytoskeleton organization and the production and reorganization of ECM molecules. The cell response to the treatment appears to predominantly involve paxillin-mediated signaling pathways. Conclusions: These changes suggest that laser treatment can potentially improve the structure and function of skin tissue, with interesting implications for treating skin aging.

## 1. Introduction

Energy-based devices and lasers are becoming increasingly common in the esthetic field, particularly for promoting rejuvenation; however, there are still gaps in users’ knowledge regarding the application of these technologies. To choose safe and effective laser treatments, it is important to know how lasers interact with tissues and cells and to understand the cellular and molecular mechanisms underlying the desired esthetic effects. Laser radiation is absorbed by chromophores present in cells and tissues. Stimulating these chromophores induces a series of biological responses that can increase the mitochondrial respiratory chain’s efficiency and release biochemical factors, such as growth factors and inflammatory mediators [1]. The mitochondrial respiratory chain’s cytochrome c oxidase (CCO), heme proteins, porphyrins, water, and melanin are among the best known and most studied chromophores [2]. The interaction between laser radiation and biological tissues can be photochemical, photothermal, or photomechanical depending on many parameters such as the emission wavelength, the tissue’s optical properties, fluence, exposure time, etc. Many lasers applied in skin rejuvenation protocols show emissions with wavelengths falling in the red or near-infrared (NIR) bands. These wavelengths can be absorbed by intracellular chromophores, such as water and CCO, affecting a wide range of biological processes in living cells and tissues, such as cytoskeleton reorganization, metabolic rearrangements, alterations in gene expression, extracellular matrix (ECM) turnover, differentiation, and proliferation [3,4,5].

As we get older, the ultraviolet (UV) component of sunlight has harmful effects on our skin over time that lead to enhanced matrix metalloproteinase (MMP) activity and collagen breakdown. Indeed, one of the main factors contributing to premature skin aging is photoaging caused by ultraviolet (UV) irradiation [6]. Deep wrinkles and texture changes are among the many alterations in the skin due to photoaging caused by increased MMP [7,8,9] and production of reactive oxygen species (ROS), which intensify oxidative stress [10]. This results in the overexpression of proinflammatory cytokines. These cytokines deteriorate the extracellular matrix (ECM), break down collagen, and form aberrant elastic fibers.

Many products and devices have been proposed to prevent, delay, and treat skin aging before cosmetic surgery is required. Among these methods, non-ablative laser treatments have been demonstrated to improve wrinkles and skin texture [11,12,13].

Collagen fragmentation induced by MMP may facilitate the synthesis of new collagen; indeed, a significant aspect of dermal remodeling is the deposition of new dermal collagen, which could partially explain the improvement observed following non-ablative resurfacing [12,14].

According to clinical evidence, a 675 nm wavelength can treat skin textures, scars, wrinkles, and pigmentations [15,16,17,18,19,20].

It was discovered that wrinkles and early aging of facial skin can be caused by variations in the normal collagen type I/III ratio. Magni and colleagues demonstrated that red light-based therapies can slow down or even stop the skin’s aging process by promoting collagen III synthesis in human adult fibroblasts in vitro [12].

Fibroblasts are the major cell population in connective tissue. They synthesize the extracellular matrix (which includes proteoglycans, fibronectin, laminins, glycosaminoglycans, metalloproteinases, prostaglandins, and collagen types I, III, and IV), thereby creating the stroma, the structural framework of animal tissues. Membrane integrins link fibroblast cytoskeletons to the ECM. Fibroblasts contribute to injury responses and repair tissue damage in both the initiation and resolution phases.

To obtain further in vitro data on the potential therapeutic benefits in rejuvenation, we employed a 675 nm laser wavelength on cultured human dermal fibroblasts to understand the mechanisms involved in the skin rejuvenation process’s signaling pathways by analyzing cytoskeletal proteins, ECM components, and membrane integrins. Changes in the expression and organization of ECM proteins are often associated with aging-related alterations in tissue structure and function [21].

By examining these proteins, we aimed to understand whether and how laser treatment could counteract the aging process at the molecular level, potentially leading to applications that can mitigate aging effects.

## 2. Materials and Methods

### 2.1. Experimental Model and Design

Normal human dermal fibroblasts (NHDFs) were used as the experimental model for this study. For their culture and maintenance, Dulbecco’s Modified Eagle Medium (DMEM) supplemented with 100 µg/mL of streptomycin, 100 U/mL of penicillin, 2 mM of glutamine, and 10% fetal bovine serum (FBS) was used. Cells were incubated at 37 °C and 5% CO_2_ and cultured in 24-well plates. In each experiment, two groups of samples were used: laser-treated fibroblast cultures and untreated controls. Each group included 6 samples. For samples designated for laser treatment, six wells were chosen in the well plate, ensuring at least one well distance between each treated well to prevent overlap of laser irradiation. The wells surrounding the treated samples were filled with cardboard to shield them from laser radiation. In each well, a coverslip (13 mm Ø) was placed prior to seeding to allow for cell adherence. A total of 25 × 103 cells were seeded per well. For the negative control samples (not receiving laser treatment), the same number of cells was seeded in six wells of a different plate.

### 2.2. Laser Source and Laser Treatment

This step only applies to cells selected for laser treatment. The laser source used in this study was a RedTouch laser (Deka Mela, Florence, Italy). Red light (675 nm) is emitted by the RedTouch laser, which uses a scanning system of 15 × 15 mm that is able to cause targeted thermal damage to the skin at a depth of 500 μm on average, reaching the dermis. Indeed, thanks to an integrated skin cooling system, it is possible to create fractional micro-zones with a 0.7 mm width (DOT) of either sub-ablative or selective thermal damage on the skin while maintaining the epidermal layer, protecting skin health and reducing downtime.

DOT pulses separated by untreated tissue heat up the region, causing collagen fibers to denature and produce new collagen [16].

For the experiment, the cells were treated using the following parameters 24 h after seeding:

- Scan time: 150 ms;
- Irradiation time: ~18 s
- DOT spacing: 500 μm;
- Power: 10 W;
- Fluence: 389.96 J/cm^2^.

In a previous study, the chosen fluence was shown to be effective in inducing collagenogenesis [12].

### 2.3. Immunofluorescence

Twenty-four hours post-treatment, the cells were fixed for 5 min in cold acetone and then washed in phosphate-buffered saline (PBS). To block nonspecific binding sites, samples were incubated with a PBS solution containing 3% bovine serum albumin (BSA) (Sigma-Aldrich, St. Louis, MO, USA) for 1 h at room temperature. Subsequently, cells were incubated overnight at 4 °C with specific primary antibodies for phalloidin, paxillin, integrin α5β1, α-SMA, vimentin, vinculin, collagen I, collagen III, laminin, fibronectin, and matrix metalloproteinase 1 (MMP1). These markers with different functions were chosen to obtain information on different mechanisms underlying fibroblast response to the laser treatment. The cells were washed three times with PBS and then incubated with fluorescein isothiocyanate (FITC)-conjugated secondary antibodies. The samples were washed again, mounted on glass slides using Fluoromount™ aqueous mounting medium, and evaluated using an epifluorescence microscope (Nikon, Florence, Italy) at 100× magnification and imaged with a HiRes IV digital CCD camera (DTA, Pisa, Italy).

### 2.4. Western Blot

For protein extraction, 50 μL of lysis solution, composed of Lysis Buffer 10× (Thermo Fisher, Waltham, MA, USA) and PMSF 100× (Thermo Fisher) in milliQ water, was added to the cell pellets. The cell pellets were then subjected to mechanical disruption by vortexing.

Protein quantification was performed using the Micro BCA™ Protein Assay Kit (Thermo Fisher, Scientific, Waltham, MA, USA), which contains a “blank” reagent (reagent A), a photosensitive reagent (reagent B), and pre-made BSA standards at 2000 μg/mL. A standard curve was generated to determine the protein concentration of the samples based on absorbance, with each point on the curve consisting of a triplicate. The curve points were observed at the following concentrations: 100 μg/mL, 80 μg/mL, 60 μg/mL, 40 μg/mL, 30 μg/mL, 20 μg/mL, and 10 μg/mL. In each well, 150 μL of solution was added with the following composition:

- Curve points: 148 μL of solution at the desired concentration + 2 μL of Lysis Buffer;
- Blank: 148 μL of H_2_O + 2 μL of Lysis Buffer;
- Samples: 148 μL of H_2_O + 2 μL of sample.

A solution with reagent A, reagent B, and reagent C in a 25:24:1 ratio was prepared. An amount of 150 μL of the solution composed of the three reagents was added to each well. The plates were then incubated for approximately 1 h at 37 °C. Finally, the absorbance values were read using a spectrophotometer at a wavelength of 490 nm. The absorbance values were used to determine the concentrations of the samples.

All the samples were prepared for the electrophoresis run as follows:

- Sample buffer 4× (Bio-Rad, Hercules, CA, USA);
- β-mercaptoethanol 10× (Bio-Rad);
- An amount of 30 μg of protein;
- MilliQ to bring each sample to the same volume.

The samples were boiled for 5 min at 95 °C and then transferred to ice for another 5 min. Finally, they were centrifuged before loading into the wells.

For the electrophoresis run and blotting, after setting up the Mini-PROTEAN^®^ Tetra Vertical Electrophoresis Cell (Bio-Rad) with the 4–20% MP TGX pre-cast Stain-Free Gel (Bio-Rad), both the outer and inner chambers were filled with cold Running Buffer (Bio-Rad). The properly prepared samples were then loaded into the gel wells, along with pre-stained protein standards, and a current of 300 V and 20–30 mA were applied. After the electrophoresis run, proteins were transferred to nitrocellulose membranes (Bio-Rad) using the Trans-Blot Turbo Transfer System (Bio-Rad). The membranes were cut according to the protein molecular weight prior to hybridization with specific antibodies. The cut membranes were blocked for 5 min with EveryBlot Blocking Buffer (Bio-Rad) and then probed with the following primary antibodies overnight at 4 °C: anti α-SMA, 1:1000 (Millipore); anti-vimentin, 1:1000; anti-tubulin, 1:1000 (Millipore, Billerica, MA, USA); anti-vinculin, 1:1000 (Millipore); anti-paxillin, 1:1000 (Millipore); anti-collagen III, 1:1000 (Millipore); anti-fibronectin, 1:1000 (Millipore); and anti-MMP1, 1:1000 (Millipore). This was followed by incubation with peroxidase-conjugated secondary IgGs (1:3000). Image acquisition and analysis were performed using Image Lab 6.1 software on a Chemi-Doc™ Touch instrument (Bio-Rad), utilizing the fluorescence emission of protein bands separated on stain-free gels for total lane normalization. * *p* < 0.05 was considered statistically significant.

## 3. Results

To reveal any morphological and functional changes induced in NHDFs by the laser treatment, an integrated approach was chosen: immunofluorescence microscopy and Western blotting were applied, allowing us to obtain qualitative and quantitative information, respectively. The parameters under study were cytoskeleton components, proteins involved in cell–ECM adhesion, and ECM molecules.

**Figure 1:**
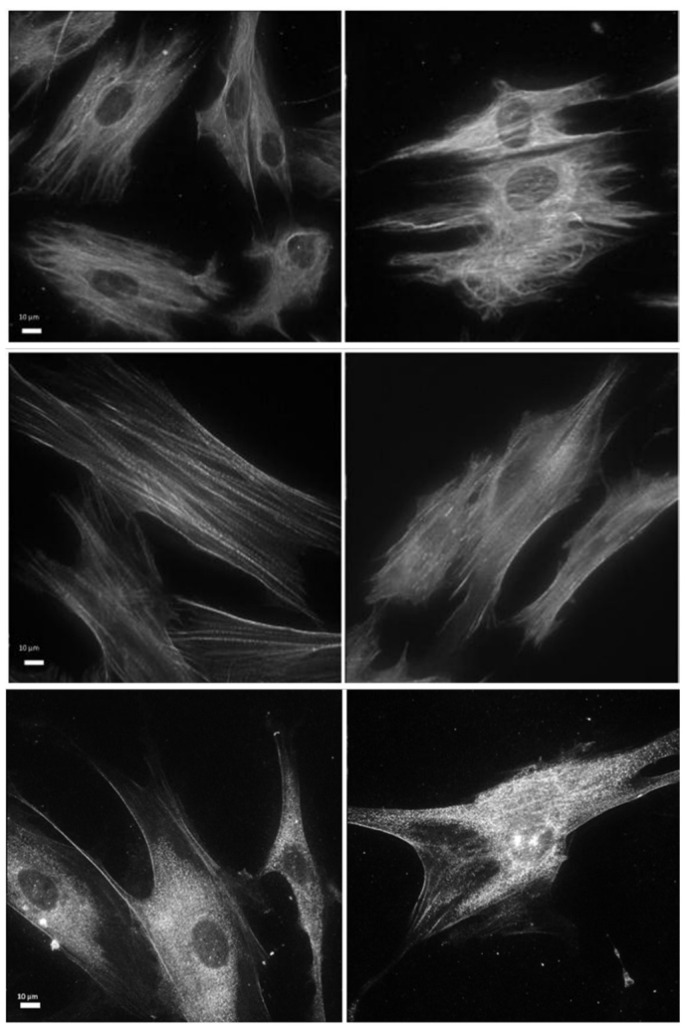
Effects of laser treatment on vimentin (**upper panels**), F-actin (phalloidin) (**middle panels**), and alpha-SMA (**lower panels**) using immunofluorescence analysis. Left: control samples; right: laser-treated samples.

Vimentin is a cytoskeletal protein that, in addition to being an important component of the intermediate filaments, helps maintain cell shape and structural integrity [22]. An immunofluorescence analysis of vimentin expression in the laser-treated NHDFs showed a denser network of cytoskeleton intermediate filaments than in the controls, suggesting a reorganization of the intermediate filaments in response to laser treatment (Figure 1 upper panels). From a quantitative point of view, the Western blot analysis did not show significant changes (*p* > 0.05) (Figure S1).

Actin filaments were stained with phalloidin, a protein that binds to actin filaments and is therefore used in immunofluorescence techniques to visualize these cytoskeletal structures. In laser-treated NHDFs, the actin filaments appeared more spread out but less thick when compared to the controls, indicating changes in actin polymerization and rearrangement of the actin filament network (middle panels in Figure 1).

α-SMA (α-smooth muscle actin), another member of the actin family, is a contractile protein involved in the contractile apparatus of smooth muscle tissue. It is also responsible for myofibroblast contraction needed for wound closure and tissue remodeling [23]. For this reason, it is considered a marker of fibroblast–myofibroblast transdifferentiation. Both immunofluorescence imaging (lower panels in Figure 1) and the Western blot analysis (Figure S2) of NHDFs exposed to laser treatment and untreated NHDFs demonstrated that the protein distribution and expression did not change significantly.

**Figure 2:**
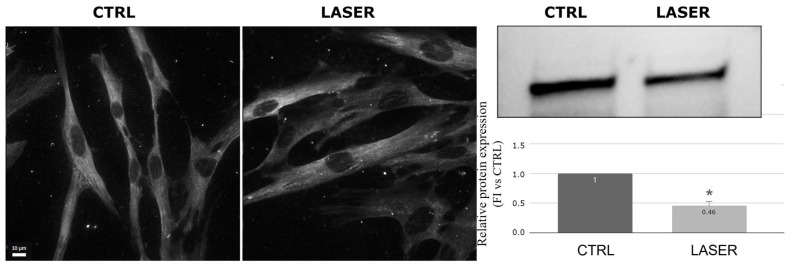
Effects of laser treatment on vinculin using immunofluorescence and Western blot analysis. Left: control samples; right: laser-treated samples. Western blot bands of control vs. laser-treated samples and graph showing the bands’ signal intensities (*p* = 0.001). The used symbol of the asterisk (*) in the figure indicates statistically significant results compared to the control, with *p* < 0.05.

Vinculin, a protein associated with focal adhesion junctions that links the actin cytoskeleton to the cell membrane, did not show significant changes in its distribution when analyzed by immunofluorescence microscopy in the laser-treated NHDFs compared to the controls (Figure 2). However, the Western blot analysis demonstrated a significant reduction in protein in the laser-treated samples (*p* = 0.001) (Figure 2).

**Figure 3:**
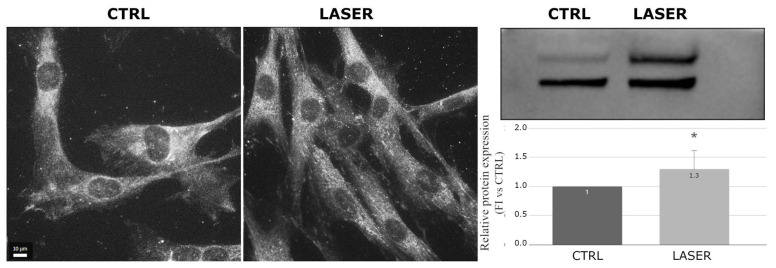
Effects of laser treatment on paxillin using immunofluorescence and Western blot analysis. Left: control samples; right: laser-treated samples. Western blot bands of control vs. laser-treated samples and graph showing the bands’ signal intensities (*p* = 0.045). The used symbol of the asterisk (*) in the figure indicates statistically significant results compared to the control, with *p* < 0.05.

Paxillin, also involved in the formation of focal adhesions and regulating actin cytoskeleton assembly and signal transduction, showed increased expression and higher spreading in the laser-treated samples imaged using immunofluorescence microscopy (Figure 3). The Western blot analysis confirmed a significant increase in paxillin levels (*p* < 0.05) in the treated samples and revealed two bands at very close molecular weights, suggesting the presence of different paxillin isoforms. Both bands showed an increase consistent with the immunofluorescence observations (Figure 3).

Collagen type I, the most abundant protein in the ECM, provides tensile stiffness and strength to connective tissues. Collagen I and the other collagen types are mostly produced by fibroblasts. In the laser-treated samples, immunofluorescence imaging and the fluorescence intensity analysis revealed decreased collagen I expression compared with the untreated controls (Figure S3).

**Figure 4:**
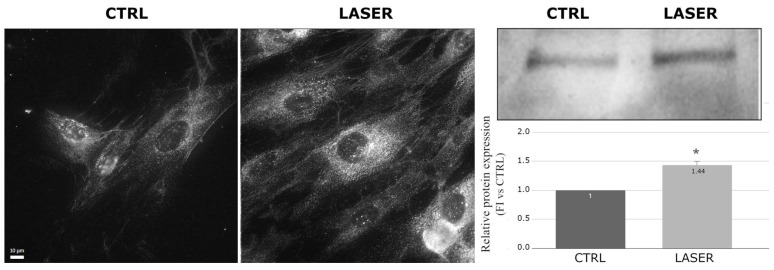
Effects of laser treatment on collagen III using immunofluorescence and Western blot analysis. Left: control samples; right: laser-treated samples. Western blot bands of control vs. laser-treated samples and graph showing the bands’ signal intensities (*p* = 0.008). The used symbol of the asterisk (*) in the figure indicates statistically significant results compared to the control, with *p* < 0.05.

On the contrary, collagen type III, a fibrillar protein that provides structural support and elasticity to tissues, including skin, showed increased expression and spreading in the cytoplasm of the laser-treated NHDFs imaged with immunofluorescence compared with the controls. The Western blot analysis also revealed a statistically significant increase (*p* = 0.008) in this protein in the laser-treated samples (Figure 4). Collagen III is also important in the response to cellular damage because it is produced in the early stages of the repair process and is crucial for the fibrillogenesis of collagen I, which increases in the late phases of repair [24].

**Figure 5:**
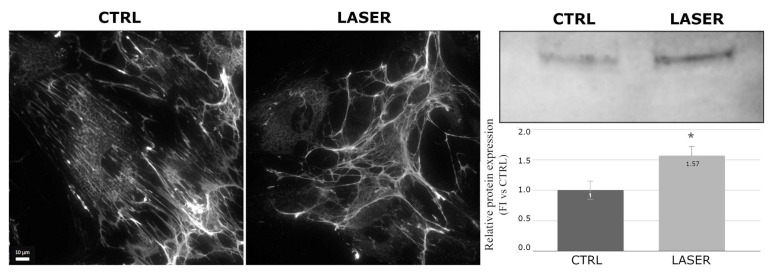
Effects of laser treatment on fibronectin using immunofluorescence and Western blot analysis. Left: control samples; right: laser-treated samples. Western blot bands of control vs. laser-treated samples and graph showing the bands’ signal intensities (*p* = 0.034). The used symbol of the asterisk (*) in the figure indicates statistically significant results compared to the control, with *p* < 0.05.

α5β1 integrin, which mediates cell adhesion to fibronectin in the ECM and plays a role in cell migration and survival, and laminin, a basal membrane protein that promotes cell adhesion, differentiation, and migration, did not show significant changes when comparing treated and untreated samples. However, in immunofluorescence imaging, the α5β1 integrin signal at the focal adhesion level appeared clearer in the laser-treated NHDFs (Figure 5). Fibrils of fibronectin, an ECM glycoprotein that promotes cell adhesion and migration and is also involved in collagen fiber formation, generated a network surrounding the cells both in the laser-treated and untreated fibroblasts. The Western blot analysis documented that fibronectin expression was significantly higher in the laser-treated fibroblasts than in the untreated ones (*p* < 0.05) (Figure 5).

**Figure 6:**
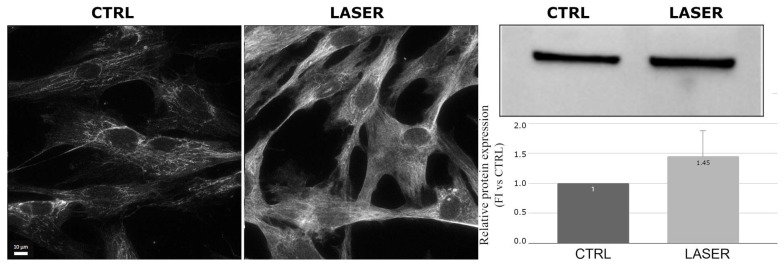
Effects of laser treatment on MMP1 using immunofluorescence and Western blot analysis. Left: control samples; right: laser-treated samples. Western blot bands of control vs. laser-treated samples and graph showing bands’ signal intensity (*p* > 0.05).

In addition to the production and assembly of matrix molecules, such as the various types of collagen, ECM turnover is regulated by the activity of metalloproteinases, which degrade matrix components, particularly collagen [25]. MMP-1, an enzyme that degrades type I, II, and III collagen and facilitates ECM remodeling, showed higher expression in the laser-treated samples than in the controls. However, the Western blot analysis did not confirm these results. Specifically, a quantitative increase in MMP-1 was observed, but it did not reach statistical significance, probably due to the wide dispersion of the data (*p* > 0.05) (Figure 6).

## 4. Discussion

In recent years, the field of esthetic medicine has seen significant advancements due in part to the development and application of laser technologies. Among these, red wavelength lasers, typically operating in the range of 600 to 750 nm, and NIR lasers, operating in the range of 780 to 1000 nm, have garnered particular attention for their unique properties and versatile applications [15,16,17,18,19,20]. The absorption characteristics of red light by biological tissues, along with its ability to penetrate deeper layers of the skin, make these lasers highly effective for a variety of esthetic treatments. Researchers are trying to understand the intricate ways in which these technologies can improve skin health and appearance by delving deeper into the cellular and molecular effects of these non-ablative laser treatments. This approach not only targets esthetic improvements but also contributes to overall tissue health. In our study, we aimed to investigate the effect induced by treatment with an IR laser source (RedTouch laser) on the organization and expression of various cytoskeletal and extracellular matrix proteins in dermal fibroblasts. These cells play a crucial role in tissue repair processes and are responsible for the deposition of a new ECM in response to damage.

Vimentin and actin are essential components of the cytoskeleton, playing critical roles in maintaining cell structure, integrity, and function [22,23]. Our results concerning vimentin expression and organization may indicate that laser treatment affects its spatial organization rather than increasing the total amount of the protein. This suggests that laser radiation might induce changes in the arrangement of vimentin within the cell, which could influence various cellular functions and structural stability. Similarly, since actin has a crucial role in cell movement, division, and intracellular transport, changes in actin polymerization and the rearrangement of the actin filament network could reflect alterations in cytoskeletal dynamics [26]. These changes are potentially related to increased cell mobility and activity induced by laser treatment. Thus, laser treatment might promote a more dynamic and responsive cytoskeletal structure, facilitating enhanced cellular processes such as tissue repair and cell migration. α-SMA is an actin isoform present in myofibroblasts and involved in tissue remodeling [27]. After laser treatment, α-SMA did not show statistically significant variations in either immunofluorescence or the Western blot analysis. This suggests that laser treatment does not significantly alter α-SMA expression, indicating that there is no relevant change in cell contraction and fibroblast-to-myofibroblast transformation, thus reducing the risk of fibrosis during the tissue repair process.

Parallel to the study of cytoskeletal proteins, focal adhesions have also been investigated. Focal adhesions are sub-cellular structures that mediate the regulatory effects of a cell in response to ECM adhesion [28]. The main components of focal adhesions are integrins, which are membrane glycoproteins that play a crucial role in linking the cell to the ECM and in transducing signals from the ECM to the cell. Observations of immunofluorescence images reveal no significant changes in the arrangement or expression of integrins. However, interesting results have been obtained through the study of proteins that interact with integrins, such as paxillin, vinculin, and fibronectin. Paxillin is a signal transduction adaptor protein that localizes to the intracellular surface of focal adhesions and binds directly to integrins. Paxillin plays a central role in coordinating the spatial and temporal actions of the Rho family of small GTPases, which regulate the actin cytoskeleton by recruiting an array of GTPase activators, suppressors, and effector proteins to cell adhesions [29]. Paxillin has been described as an important player in skin fibroblast morphology, and it has been shown that its levels decrease notably during the aging process, consequently leading to reductions in collagen I production and fibroblast contractility [30]. Paxillin also has a crucial role in the system that transduces the signals deriving from the cell–matrix interaction. It is well known that fibroblasts regulate matrix turnover by producing metalloproteinases and ECM components, but the ECM regulates fibroblast morpho-functional phenotypes through biochemical and mechanical cues. An altered cell–matrix interaction due, at least in part, to a decrease in paxillin expression has been associated with aging, and it has been suggested that restoring the paxillin-mediated cell–matrix interaction might play a significant role in anti-aging actions [31]. Our analysis shows that paxillin levels increased significantly after the laser treatment, suggesting that laser radiation could help reverse the decrease in paxillin levels during the aging process.

Vinculin consists of a head domain and a tail domain connected by a flexible linker, enabling it to switch between open and closed conformations. The head domain of vinculin shares several key binding partners and functions with paxillin, with paxillin itself being one of these partners. However, unlike paxillin, which does not directly interact with actin, the tail domain of vinculin can bind directly to actin filaments and interact with actin-binding proteins such as α-actinin [32]. In the literature, a reduction in vinculin has been associated with increased cytoskeletal plasticity and cell motility [33]; this may facilitate cellular remodeling processes and more dynamic responses. The reduction in vinculin observed in our analyses could indicate a change in the dynamics of focal adhesions and in the interaction between the cytoskeleton and the ECM.

Fibronectin is an essential glycoprotein for cell–matrix adhesions as it can bind both integrins on the external side of the plasma membrane and ECM molecules, such as collagen. Recent studies in the literature show that rejuvenated fibroblasts exhibit increased matrix protein deposition, including fibronectin [34]. In agreement with the current literature, our results show a significant increase in fibronectin expression after laser treatment. It is well established that fibronectin, together with other proteins such as heparan sulfate, acts as a scaffold to organize enzymes and substrates for procollagen processing [35]; therefore, we decided to investigate collagen expression.

While the analysis of collagen I expression showed a slight reduction after the laser treatment, a significant increase in collagen III expression was observed. These data should not be seen as contradictory; they can be explained by the fact that the production of collagen I and III occurs at different stages of tissue repair. Initially, collagen III synthesis predominates, which is later replaced by collagen I [36]. As shown in our experiments, the early increase in collagen III induced by the non-ablative laser treatment, along with the slight reduction in collagen I, suggest potential reorganization and increased deposition of the ECM, which may enhance the structure and resilience of skin tissue, therefore confirming its anti-aging effect in the esthetic field. The results confirm the outcomes of a preliminary study [12] and demonstrate the anti-aging effect of the laser source and treatment protocol applied in this study.

Finally, the expression of MMP-1, also known as interstitial collagenase, was evaluated because its main targets are collagen types I, II, and III [37]. Although not reaching statistical significance, the trend toward increased MMP1 expression further supports the hypothesis that laser treatment promotes the reorganization of the matrix and remodeling of collagen fibers.

We conducted a cellular investigation to understand the molecular mechanism involved in the skin rejuvenation process’s signaling pathways in accordance with clinical evidence that has already been tested. In fact, in previous investigations, we showed that the RedTouch laser’s capacity to interact with collagen fibers makes it a potentially effective therapy for chrono aging [15,16,17]. In these clinical articles, we compared our non-ablative laser system with other gold-standard treatments for rejuvenation. Ablative lasers vaporize tissue and are therefore more aggressive than non-ablative lasers. Compared to ablative lasers (CO_2_/erbium lasers), non-ablative lasers have much fewer possible harmful consequences, and their main advantage is a significant decrease in postoperative downtime.

### 4.1. Potential Future Applications

The RedTouch device emits a laser in the visible spectrum, and a fractional scanning system allows micro thermal zones (with an average depth of 300/500 microns) to be generated; this thermal column conducts heat to the surrounding areas, causing immediate collagen shrinkage and denaturation with subsequent new collagen formation. Laser-generated thermal columns could positively impact wound healing through antibacterial and anti-inflammatory properties (in addition to neocollagenesis), promoting the release of cytokines and growth factors that are crucial for wound management [37,38,39].

Injectable hyaluronic acid fillers and laser/light procedures have become increasingly popular for non-invasive facial rejuvenation in many cosmetic practices. As a result, in a preclinical internal laboratory test, we have already examined the possible interaction between the study device and some hyaluronic acid-based fillers using a Raman spectroscopy analysis, showing that the tested device is safe when interacting with hyaluronic acid without changing its molecular structure. From a clinical approach, a 675 nm laser interacts with hyaluronic acid-based fillers to improve its effectiveness in skin rejuvenation procedures (data are in the process of publication). Natural polysaccharide hyaluronic acid, which is generated by fibroblasts during the wound repair stages of proliferation, can mediate cellular signaling, encourage cell migration, and induce morphogenesis and the matrix structure, all of which improve the soft tissue wound healing process [40].

A hydrogel is an innovative medical device that promotes healing due to the presence of hyaluronic acid, protecting the wound by promoting skin re-epithelialization.

Since existing studies indicate that NIR irradiation enhances hydrogels’ in vivo antibacterial properties [41,42,43], we may consider applying our technology to the field of wound healing with a completely new approach; this is one of our next research goals.

### 4.2. The Limitations of This Study

A limit of this study is that it considered a single treatment, the effects of which were analyzed after 24 h, i.e., at a single time point. Based on the results, further investigations considering repeated treatments and several post-treatment timepoints will be implemented in future research.

## 5. Conclusions

In conclusion, the results from immunofluorescence and the Western blot analyses indicate that the laser treatment evaluated in this study induces significant changes in human dermal fibroblasts, particularly affecting cytoskeletal organization and the production and reorganization of extracellular matrix (ECM) molecules. The cellular response to this treatment is closely associated with paxillin-mediated signaling pathways, with paxillin acting as a key adaptor protein in focal adhesions and playing an essential role in cell–matrix interactions. The significant increase in paxillin levels observed after laser exposure suggests that this treatment may counteract the decline in paxillin levels associated with aging, potentially benefiting fibroblast morphology, contractility, and ultimately skin structure. This effect may influence matrix turnover and tissue remodeling, thus providing an anti-aging action further corroborated by increases in fibronectin and collagen III, which are both involved in the early stages of tissue repair.

Overall, these findings highlight the potential of laser treatment as a safe and less invasive clinical approach to skin rejuvenation, with meaningful benefits for patient well-being and quality of life. However, further in-depth studies will be necessary to explore the molecular mechanisms underlying these effects and to validate the anti-aging efficacy of this treatment.

## Data Availability

The data that support the study’s findings are available upon reasonable request from the corresponding author.

## Supplementary Materials

The following supporting information can be downloaded at: https://www.mdpi.com/article/10.3390/biomedicines12122713/s1, Figure S1. Laser emission at 675 nm: molecular counteraction of the aging process; Effects of laser treatment on Vimentin using Western blot analysis. Western blot bands of control vs. laser-treated samples and bands’ signal intensity graph (*p* > 0.05). Figure S2. Laser emission at 675 nm: molecular counteraction of the aging process; Effects of laser treatment on Alpha-SMA using Western blot analysis. Western blot bands of control vs. laser-treated samples and bands’ signal intensity graph (*p* > 0.05). Figure S3. Laser emission at 675 nm: molecular counteraction of the aging process; Effects of laser treatment on Collagen I using immunofluorescence and Fluorescence Intensity analysis. On the left: control samples: on the right laser-treated samples. Fluorescence Signal Intensity graph of control vs. laser-treated samples (*p* < 0.05).
