# Supplementary material for: Laser Emission at 675 nm: Molecular Counteraction of the Aging Process
Source: Biomedicines. 2024 Nov 27;12(12):2713. doi: 10.3390/biomedicines12122713 (PMC11673938; doi:10.3390/biomedicines12122713)
Supplement: Supplementary file 1 [file biomedicines-12-02713-s001.zip › biomedicines-3293510-supplementary.pdf]

biomedicines

MDPI

Supplementary Materials

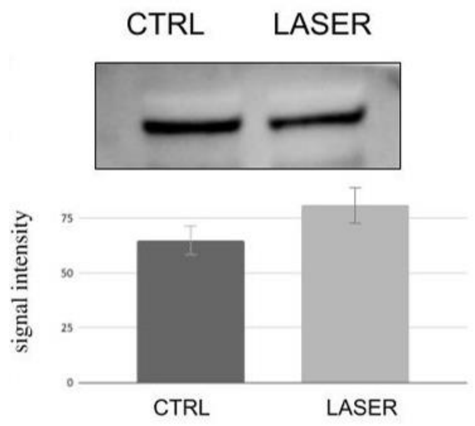

Figure S1. **Laser emission at 675 nm: molecular counteraction of the aging process**; Effects of laser treatment on Vimentin using Western blot analysis. Western blot bands of control vs. laser-treated samples and bands' signal intensity graph ($p > 0.05$).

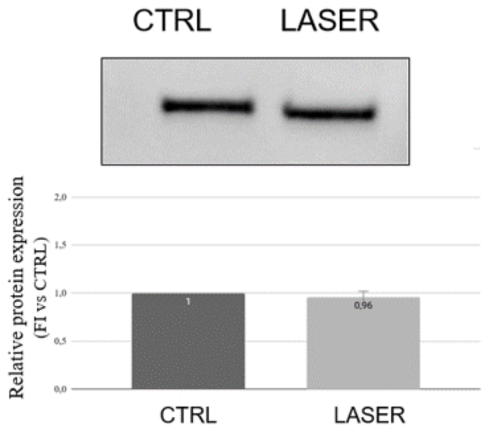

Figure S2. Laser emission at 675 nm: molecular counteraction of the aging process; Effects of laser treatment on Alpha-SMA using Western blot analysis. Western blot bands of control vs. laser-treated samples and bands' signal intensity graph ($p > 0.05$).

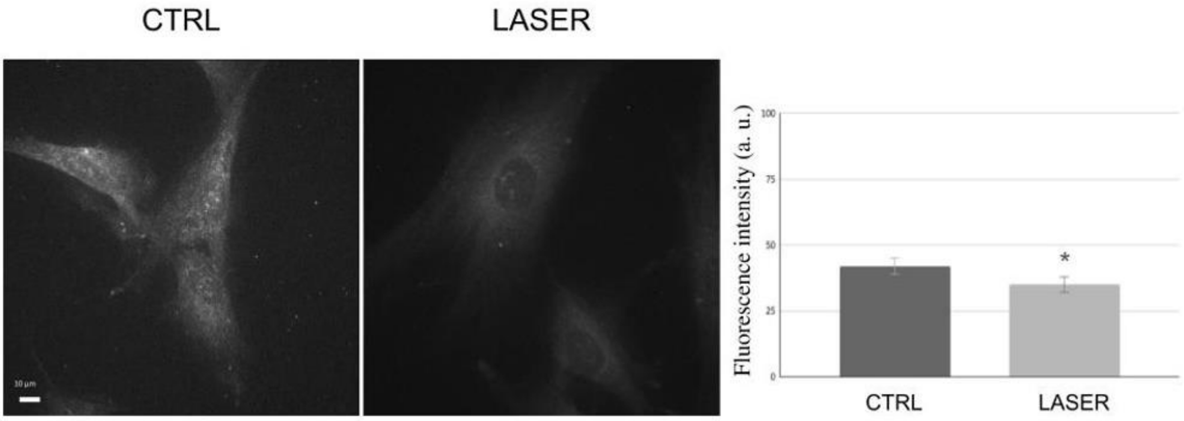

Figure S3. Laser emission at 675 nm: molecular counteraction of the aging process; Effects of laser treatment on Collagen I using immunofluorescence and Fluorescence Intensity analysis. On the left: control samples: on the right laser-treated samples. Fluorescence Signal Intensity graph of control vs. laser-treated samples ($p < 0.05$).
